# Mechanical behaviour of thick flax and carbon-flax hybrid laminates under multiple load cases

**DOI:** 10.1177/07316844261435063

**Published:** 2026-03-16

**Authors:** Scott L. J. Millen, Michael Shaw, Scott Duckhouse, Rifat Wahid, Emer McAleavy

**Affiliations:** 1 School of Mechanical and Aerospace Engineering, 1596 Queen’s University Belfast, Belfast, Northern Ireland; 2 Unit 1 Oakbank, Artemis Technologies Limited, Belfast, Northern Ireland

**Keywords:** natural fibre composites, mechanical testing, open-hole tensile strength, hybrid composites, interlaminar shear strength (ILSS)

## Abstract

The potential of natural fibres as a replacement reinforcement in traditional fibre reinforced polymer composite applications has seen increasing investigation due to their cost and environmental impact. Thick composites are also being investigated for both structural and tooling applications during manufacturing. This work assessed the structure-property relationships governing failure in thick (∼10 mm) flax fibre reinforced polymer (FFRP) laminates and 1-8-1 hybrid laminates with Carbon Fiber Reinforced Polymer (CFRP) facesheets, across different loading modes, using unnotched tensile, combined loading compression (CLC), open-hole tensile (with Digital Image Correlation), flexural and interlaminar shear strength (ILSS) tests. Post-mortem visual and optical observations were made using a microscope and scanning electron microscopy (SEM). The results provide insight into the mechanical behaviour of these laminates and the influence of carbon face-ply hybridisation. Hybridisation selectively enhances stiffness- and interface-dominated properties (flexure, ILSS), while exerting minimal influence on net-section tensile and OHT strength. Results were consistent among tests, with carbon-flax and flax-only laminates exhibiting comparable net-section tensile strengths. The unnotched tensile strengths differed by less than 1%, indicating that hybridisation does not significantly alter tensile strength in this laminate configuration. The addition of carbon-fibre facing plies had a modest effect on strengths except for ILSS and flexural strength which increased by 29% and 66%, respectively.

## Introduction

The application of flax fibre reinforced polymer composites (FFRP) is increasing in many structural applications,^1,2^ due to their environmental benefits^3,4^ and despite potential concerns including moisture retention.^5^ For example, a study considered the use of flax as a structural component in bicycle frames, based on potentially superior vibration damping characteristics.^6,7^ Amiri et al.^6^ used 70% flax to 30% carbon fibre, and found the frame had a comparable stiffness to carbon fibre only, was lighter than aluminium and had better vibration damping than all common frame materials.

Studies have assessed the mechanical performance of FFRP specimens using different experimental tests. However, most characterisation efforts have focused on thin laminates (typically 1–5 mm), with fewer studies examining thick laminates relevant to tooling, marine structures or highly loaded compression-dominated components. Studies have determined the flexural performance of: woven flax-reinforced epoxy composites,^8^ flax-based composites reinforced with carbon fibres^9–11^ and short flax fabric-reinforced epoxy tubes.^12^ Flexural studies on woven flax laminates have considered differing thickness from 3.32 mm^8^ up to 9.9 mm^13^ and also bending fatigue.^14^ A number of studies have assessed FFRPs under tensile loading with thicknesses ranging from 1 to 3 mm^15–17^ and open hole tension at 5 mm thick.^18^ Cihan et al.^19^ assessed the mechanical and dynamic performance of woven flax/E-glass hybrid composites while Rakesh et al.^20^ assessed drilled-hole failure in glass fibre reinforced plastics. In addition, studies have considered the fatigue behaviour of hybrid flax-glass/epoxy composites^21^ and flax/PLA composites^22^ and the bearing performance of flax fibre reinforced epoxy composites.^23^ Mode I interlaminar fracture toughness has also been assessed using double cantilever beam (DCB) testing^13^ while translaminar fracture toughness of unidirectional flax/epoxy composite has been studied.^24^ Relatively limited study of FFRPs has been completed under compression, for example, Bambach^25^ tested flax-based composite plates and channel sections in pure compression.

Other studies have considered biaxial testing^26^ and extreme loading on flax-based structures. After initial characterisation, Gabriel et al.^13^ studied the blast behaviour of FFRP laminates up to 9.9 mm thick while Wang et al.^27^ assessed the mechanical, low-velocity impact, and hydrothermal ageing properties of flax/carbon hybrid composite plates.

As noted earlier, the thickness of test specimens has varied between studies. Batouche et al.^28^ conducted a dedicated study on flax/epoxy composite laminates, considering bending tests and interlaminar shear tests with Acoustic Emission and the Digital Image Correlation (DIC) techniques, post-test Computed Tomography (CT) scans and scanning electron microscopy (SEM). Specimens were 8.32 mm thick with 64 layers and the observed failure modes suggested gradual failure, but initiation occurred earlier and propagated more rapidly than thin flax/epoxy laminates. In addition, more brittle failure was recorded for thick flax/epoxy laminates. This brittleness could be attributed to reduced ability of the laminate to dissipate energy through interlaminar shear and delamination mechanisms leading to more rapid damage propagation. These observations suggest that laminate thickness results in mechanical behaviour not directly scalable from thin-laminate data.

Hybridisation of laminates, natural-synthetic hybrids^29^ or the use of both flax and carbon fibres, have been investigated as an effective way to enhance the mechanical and hydrothermal resistance of flax-reinforced polymer composites.^11,27,30^ Wang et al.^27^ compared 5F, 5C, CFFFC and FCFCF specimens (where F is flax and C is carbon-fibre) with thicknesses of 1.5–2.5 mm. Al-Hajaj et al.^31^ studied the impact performance of woven carbon fibres/UD flax fibres and woven carbon fibres/cross-ply flax fibres and found both had superior impact properties compared to pure flax specimens. Although prior work reports improved flexural performance and impact tolerance in hybrid laminates, the literature rarely evaluates how hybridisation influences multiple load cases on a single, consistent laminate architecture. Furthermore, the effect of hybridisation on strain localisation around open holes, relevant to joint design, remains insufficiently studied, particularly in thick composites.

In summary, a number of studies have assessed individual strength and stiffness properties of various FFRP composites, and some of these have focussed on thick laminates, up to and including 9.9 mm. Other studies have assessed the effect of hybridisation with the inclusion of glass or carbon layers. To date, most studies on thick flax laminates have examined only one or two mechanical properties or relied on varying laminate structures, making cross-comparisons difficult. Consequently, the underlying structure-property relationships governing failure in thick flax laminates across different loading modes are still not well-understood. The key contribution of this work is a comparative assessment of flax and carbon-flax hybrid laminates by performing a range of tensile, open-hole tensile (with DIC), flexural, interlaminar shear (ILSS) and combined loading compression tests on thick flax and carbon-flax hybrid laminates. Furthermore, the near-hole strain field comparison between thick flax and carbon-flax hybrids using DIC provides new understanding of how hybridisation influences local deformation in thick laminates. Finally, new insight into how outer carbon plies affect failure modes and stiffness in thick natural-fibre laminates is provided.

## Methodology

### Test specimen manufacture

Test specimens for mechanical testing were manufactured using hand lay-up with vacuum bag resin infusion. Plies were cut into 300 mm × 200 mm rectangles and stacked on flat aluminium tooling plate. Two different laminates were manufactured, pure flax with nine plies and a 1-8-1 flax-carbon hybrid with a core of eight flax plies and one carbon ply on each face, as shown in Figure 1. The thickness of both laminates was approximately 10–11 mm.

**Figure 1.**
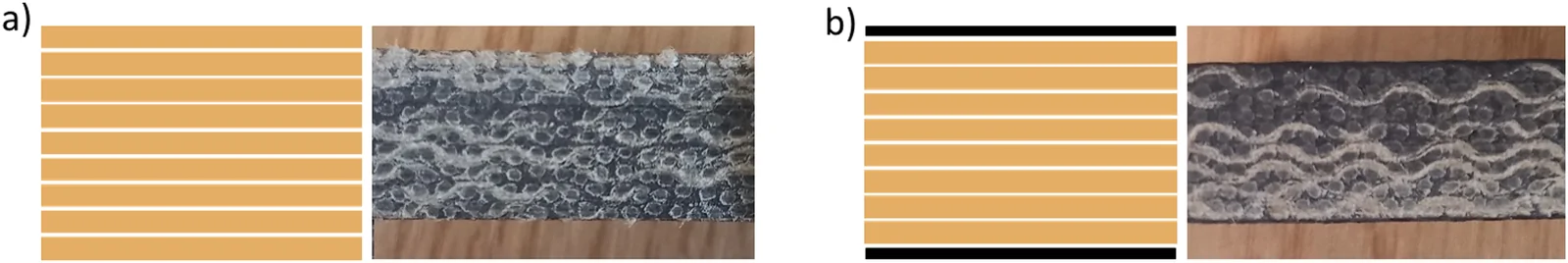
Visual representations of (a) pure flax and (b) flax-carbon hybrid laminates with cross-sectional photographs.

550 g/m^2^, 2 × 2 twill weave flax fibres were used with IB2 Epoxy Infusion Bio Resin, a low viscosity resin optimised for high performance infusions with 38% plant derived content. In the case of hybrid laminates, 200 g/m^2^ 2 × 2 twill woven carbon fabric was also used. A twill weave architecture was selected rather than a plain weave because twill fabrics exhibit lower crimp and improved drape, which is advantageous in the manufacture of thick laminates. Lower crimp reduces fibre waviness and helps maintain more uniform load transfer between plies, while improved drape reduces the formation of resin-rich pockets that can occur when stacking many layers of plain-weave fabrics. The influence of fibre density and tow direction within the woven structure was not the primary focus of this study. However, the adoption of a symmetric and balanced lay-up ensured that directional effects did not bias the global laminate response. As such, the mechanical behaviour reported reflects the laminate-level performance rather than variations attributable to fabric architecture.

Approximately 700 ml of resin was added to a mix pot before the required amount of hardener was calculated using the specified ratio of 100/22 g resin-hardener and added to a separate container. The hardener was slowly added to the resin while stirring in a fume cupboard. The mixture was stirred for a further 2–3 min after all hardener was added. The resin mixture was placed in a de-gassing chamber, a vacuum was pulled and left for 5–10 min until bubbles had dissipated.

Peel ply, perforated release film and breather fabric were placed on top of the dry fabric layers and sealed with vacuum bag under a pressure of −1 bar, as shown in Figure 2. Peel ply was cut approximately 40 mm larger than the test laminate. Flow mesh was cut to approximately 50 mm larger than the laminate, then a semi-circle was cut from the outlet end. Every edge of the mesh was taped down using green stick tape, to prevent the puncture of the vacuum bag. The vacuum bag was cut to size approximately 50 mm larger than the tool, placed over the tool and laminate and carefully attached around all sides.

**Figure 2.**
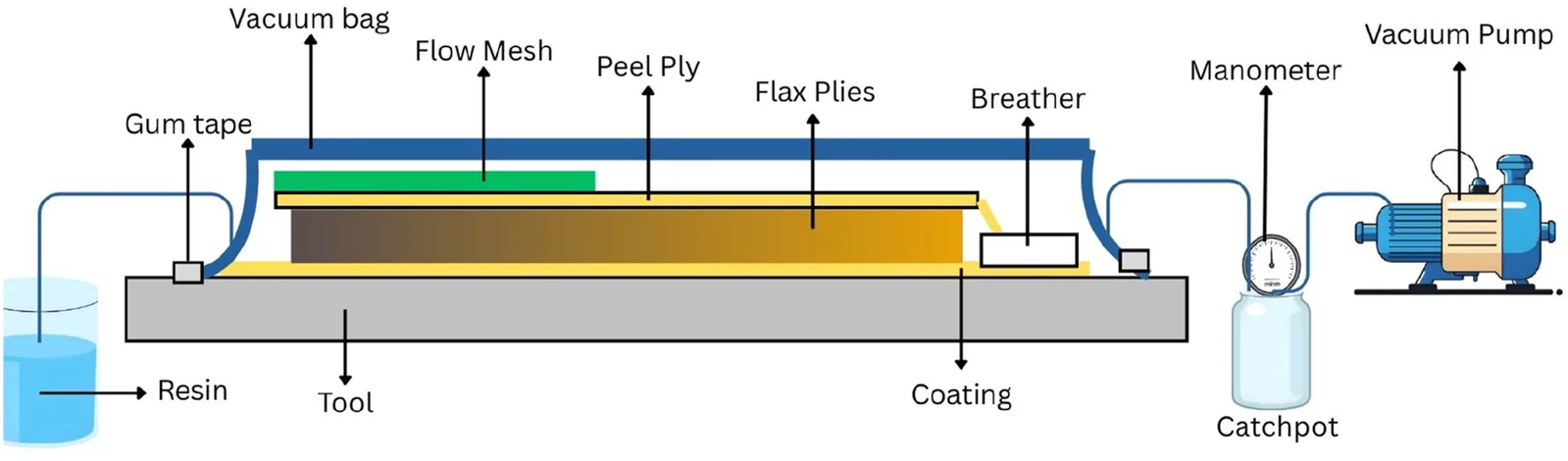
Diagram of infusion materials and laminate.

The infused laminates were left to cure for 24 h at room temperature (∼22°C) with a further 24 h at 40°C in an oven. After the curing process, all consumables were separated from the laminate which was then inspected. Infusion quality was monitored visually, and no dry-spots or large voids were observed in the panels. The nominal fibre volume fraction was approximately 45 ± 3%. In order to further confirm the infusion quality four small samples were cut from different infused panels. These were mounted in 3D printed Polylactic Acid (PLA) jigs for polishing (Figure 3(a)), and to allow scanning for voids through the 10 mm thickness of the specimens. Samples were then polished using a Buehler EcoMet 30 grinder polisher with silicon carbide disks and water coolant. Given the samples were soft, polishing was completed in a short timeframe and once polished, samples were imaged using a Nikon SMZ800 microscope (Figure 3(b)). In order to quantify the void content, images were analysed using high contrast and colour saturation to highlight the voids (Figure 3(d)), then a pixel count was completed to quantify the void percentage in each cross-section. Samples two and three had the highest void percentages of 1.87% and 1.57%, respectively. While 1–2% is relatively high for a structural component, this is an acceptable range for an infused flax material and compares well with other studies who found 1.7%–2% void content in thinner, 3 mm laminates.^32,33^ The low specimen-to-specimen variability in mechanical results also suggests consistent laminate manufacture.

**Figure 3.**
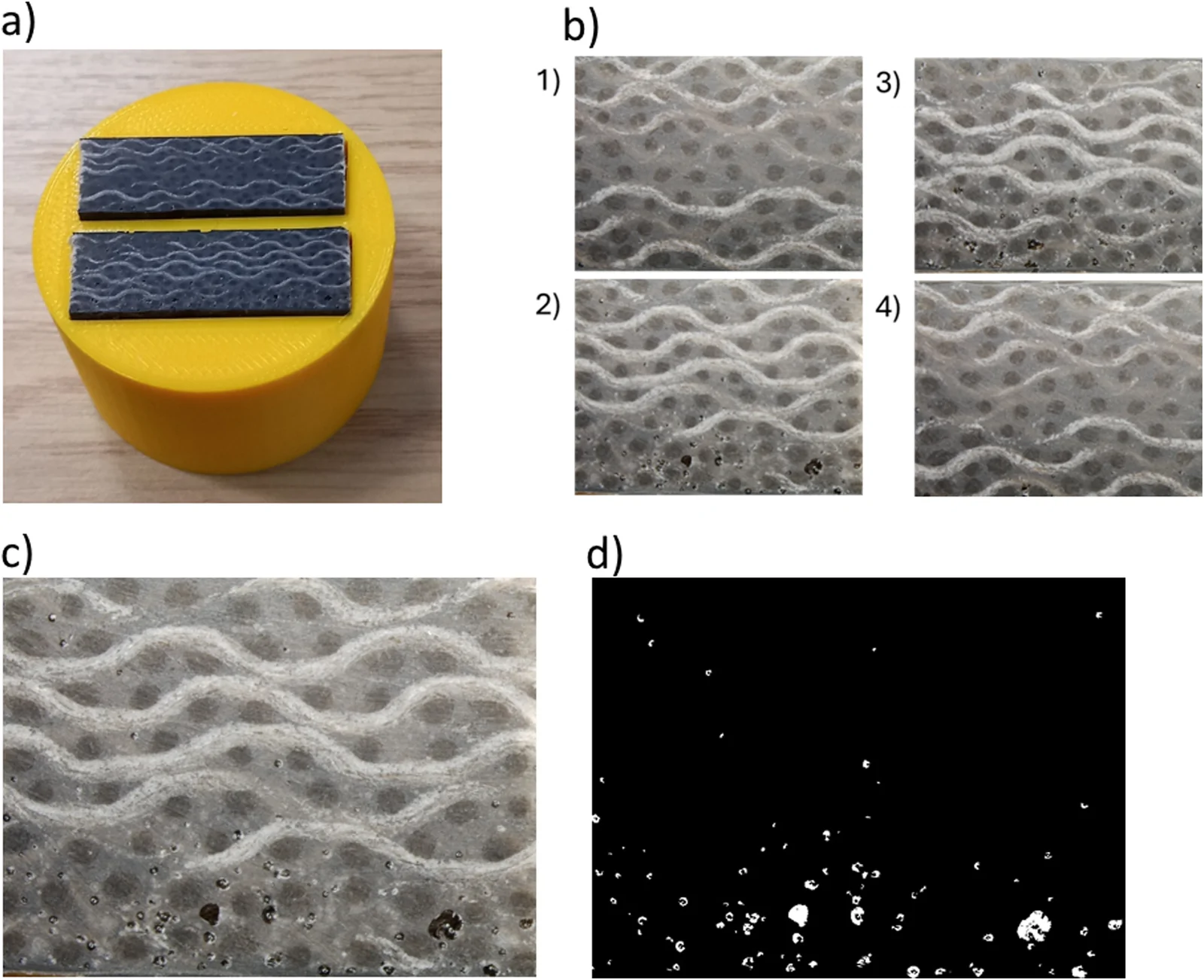
Images of (a) flax samples mounted for polishing, (b) sample cross-sections showing voids, (c) raw microscope image of void sample two and (d) voids isolated in sample two using image modification techniques.

Test specimens were cut using a STEPCRAFT 600 CNC machine to the dimensions according to each mechanical testing standard. The length, width and thickness of each specimen was recorded before the start of each test. Test specimens had an average thickness of 10.36 mm with a standard deviation of 0.28.

### Mechanical testing

All testing was completed using a Zwick Roell Z100 universal testing machine, Figure 4, with a 100 kN load cell for tensile, combined loading compression (CLC) and open-hole tensile and 10 kN for flexural and interlaminar shear strength (ILSS). Six types of testing were completed, tensile, compressive, flexural, interlaminar or short-beam shear and open-hole tensile, according to specific test standards:

• ASTM-D3039 [Tensile],
• ASTM-D6641 [Combined Loading Compression],
• ASTM-D790 [Flexural],
• ASTM-D2344 [Interlaminar shear strength],
• ASTM-D5766 [Open-hole tensile],

**Figure 4.**
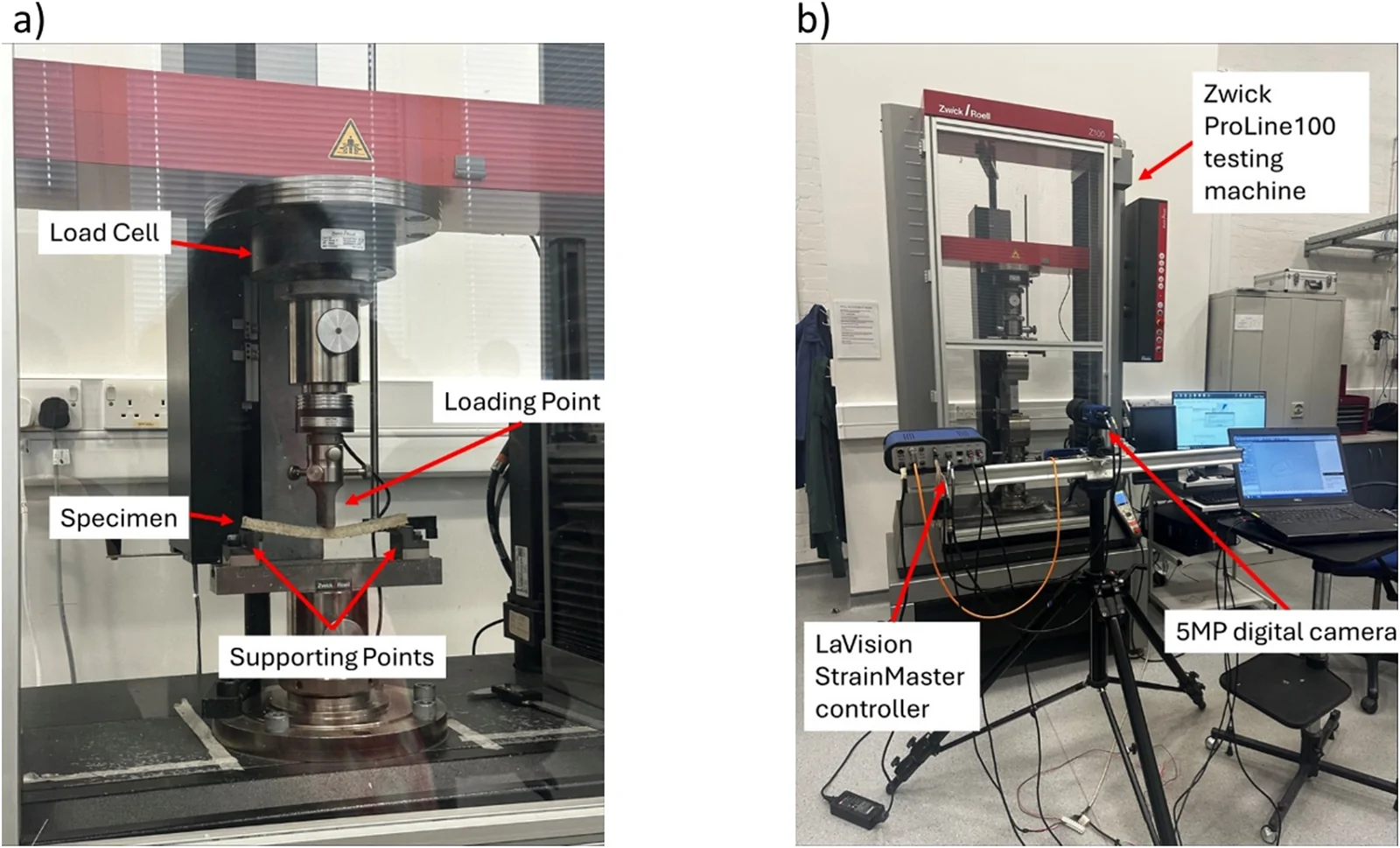
Zwick Roell Z100 universal testing machine setup for (a) flexural testing and (b) OHT with DIC.

#### Unnotched tensile testing

Unnotched tensile testing was completed at a speed of 2 mm/minute and with a pre-load of 100 N.

#### Combined loading compression (CLC) testing

CLC testing was completed at a speed of 1.3 mm/minute with a pre-load of 150 N. The specimen was placed into the combined loading compression test fixture (ASTM-D6641), fixture number CU-EL-91 and secured using a number of bolts. All specimens were compressed until maximum force was achieved and were then further compressed until pure failure, approximately 10 mm before fixture contact.

#### Flexural and ILSS testing

The flexural testing attachment, using a three-point bending configuration, was set to maximum span of 160 mm (Figure 4(a)), while ILSS testing had a distance between supports of 50 mm. Both flexural and ILSS testing were completed at a speed of 2 mm/minute with a pre-load of 1 N. The flexural strength was calculated based on the maximum load measured during testing.

#### Open-hole tensile testing

Open-hole tensile testing was completed with a loading rate of 2 mm/min. Each open-hole tensile (OHT) specimen was drilled using a STEPCRAFT 600 CNC to a diameter of approximately 4 mm. This was then increased using a hand held drill to a diameter of 6 mm. Loading was applied until a significant drop in load was observed or the specimens failed catastrophically. Each OHT test was completed with Digital Image Correlation (DIC) to investigate non-linear strain distribution around the hole. Prior to testing each OHT specimen was sprayed with a thin layer of white paint. A speckle pattern was then added to each sample using an ink stamp from correlated solutions. The speckle size was measured across samples and the diameter of speckles was found to vary between 147.06 µm and 294.12 µm with an average speckle diameter of 220.59 µm. An LaVision Imager E-lite 5MP digital camera was positioned on a tripod at a fixed distance from the test rig (Figure 4(b)), perpendicular to the plane of the sample, to capture two-dimensional images of the speckle pattern and assess in-plane deformations. Images were captured at an acquisition rate of 1 Hz from initial loading to specimen failure. LaVision’s DaVis 8.4 software was used for image analysis. A subset size of 29 with a step size of 8 pixels was chosen. The full-field in-plane strain distribution could be estimated by comparing deformed and un-deformed images. During OHT testing and DIC acquisition, load data from the Zwick ProLine100 testing machine was accessed via an input–output interface card. This analogue voltage signal passed through an analogue-to-digital converter in the LaVision StrainMaster controller. Individual image frames and load measurements could then be synchronised. Five repeats were tested for each laminate structure.

A custom Matlab script, developed by Hawkins et al.,^34^ was used for post-processing with ReadIMX and PIVMAT Matlab toolboxes. Using this script, full-field strain images could be extracted at defined stress levels. The average absolute strain was calculated at different distances from the hole using an approach of concentric circles. The median strain from all of the specimens tested for each laminate was plotted against distance from the hole. An example is shown in Figure 5.

**Figure 5.**
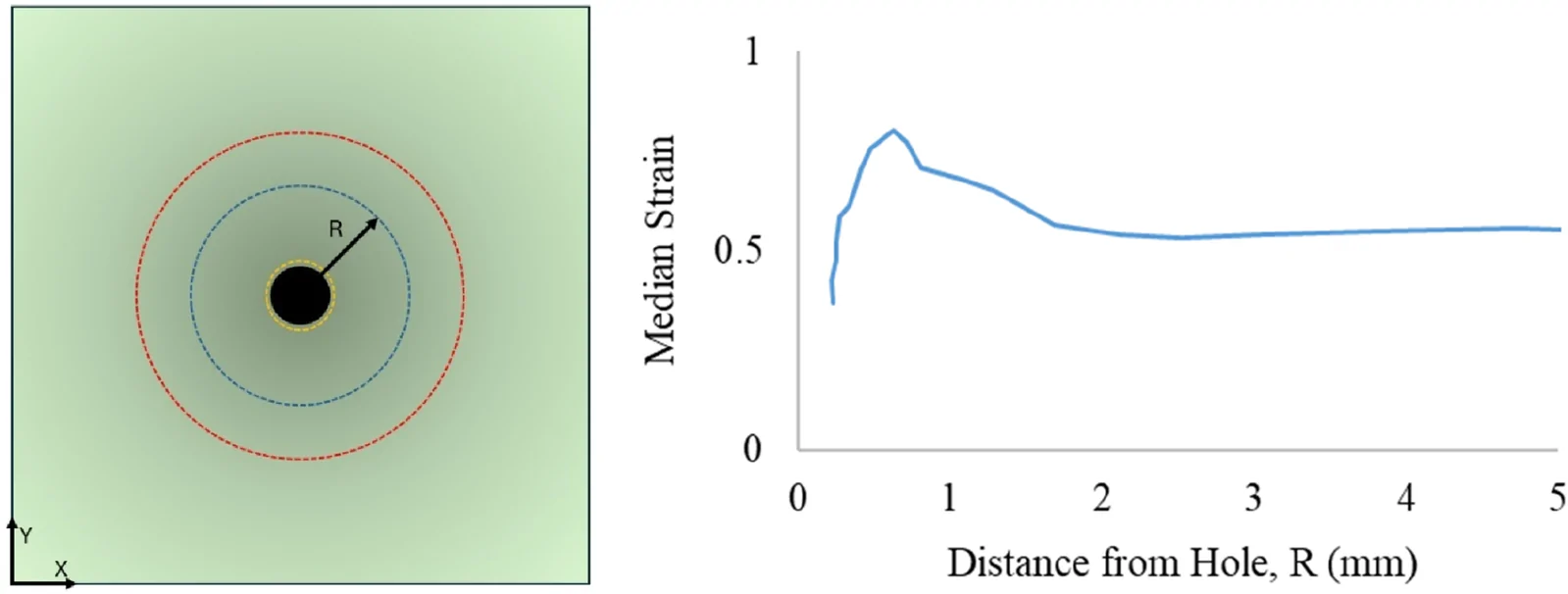
Exemplar average strain extraction across specimens at distance, R, from the specimen hole.

### Visual and optical assessment

#### Microscope

A Nikon SMZ800 microscope was used to assess the failure zone post-tests. A magnification of 2x was used with images captured for each material system and annotated using a scale bar. Regions analysed included ILSS failure planes and tensile failure surfaces.

#### Scanning electron microscope (SEM)

Flax samples were prepared via a water fed circular saw for SEM scanning. The samples were fixed via double side tape to an appropriate stub size (52 mm), the height was adjusted manually and clearance was tested using a gauge. The Hitachi flexSEM1000 with a magnification of x65 and x75 was used and the roller ball was used to adjust X and Y to the desired scan position and a slow scan speed was selected. The positions identified were the failure during ILSS and the failure surface after tensile testing.

## Results

### Mechanical test results

Mechanical testing results for ILSS, OHT, CLC, flexural and unnotched tensile testing are shown in Figure 6. Across all specimens, results were consistent among tests, that is, carbon-flax strengths were higher than flax only. However, the unnotched carbon-flax specimens had a lower tensile strength than their flax counterparts.

**Figure 6.**
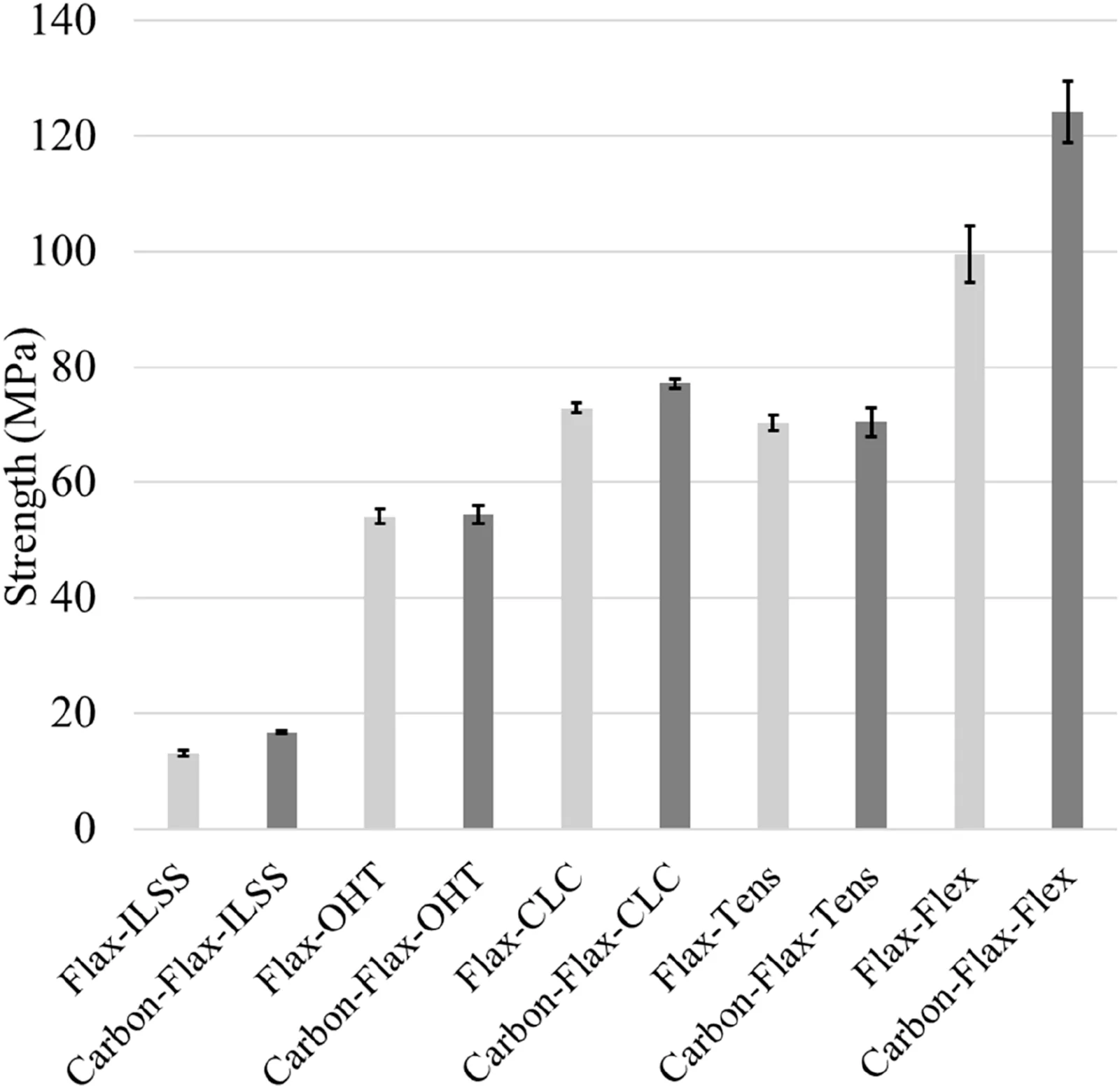
Mechanical testing results for ILSS, OHT, CLC, unnotched tensile and flexural testing for both flax and carbon-flax laminates.

### ILSS results

The ILSS for each specimen was calculated using equation (1):

(1) $$F^{sbs}=0.75\times \frac{P_{m}}{b\times h}$$

where $F^{sbs}$ is the short-beam strength (MPa), $P_{m}$ is the maximum load during the test (N), and b and h are the measured specimen width and thickness, respectively (mm). The average ILSS for the flax specimens was 13 (±0.5) MPa while the addition of carbon facing plies increased this by 29% to 16.8 (±0.3) MPa. Figure 7 compares the failure modes in the short beam tests for both flax and carbon-flax specimens observed post-test using a microscope and shows distinctly different failure paths. Failure in both laminates initiated through tension and propagated diagonally in the carbon-flax specimens, reflecting redistribution of shear stresses due to the stiffer carbon face plies, inducing interlaminar failure concentrated near the carbon/flax interface, but propagated vertically through the flax specimens, consistent with transverse tensile rupture of tow bundles and lower resistance to interlaminar shear. This difference arises because stiffer carbon plies carry axial load, increasing interlaminar shear transfer into the adjacent flax plies. This elevates shear stresses near the interface, promoting diagonal/slanted shear cracking rather than vertical tow splitting. Additionally, carbon face plies constrain out-of-plane deformation, producing cleaner fracture surfaces and reduced fibre tufting. Figure 7(b) and (d) compare the bottom surface of the specimens post short beam tests. It can be seen that the flax specimen exhibits tufting and protruding fibres. The addition of carbon plies constrains this effect and produces a cleaner failure surface.

**Figure 7.**
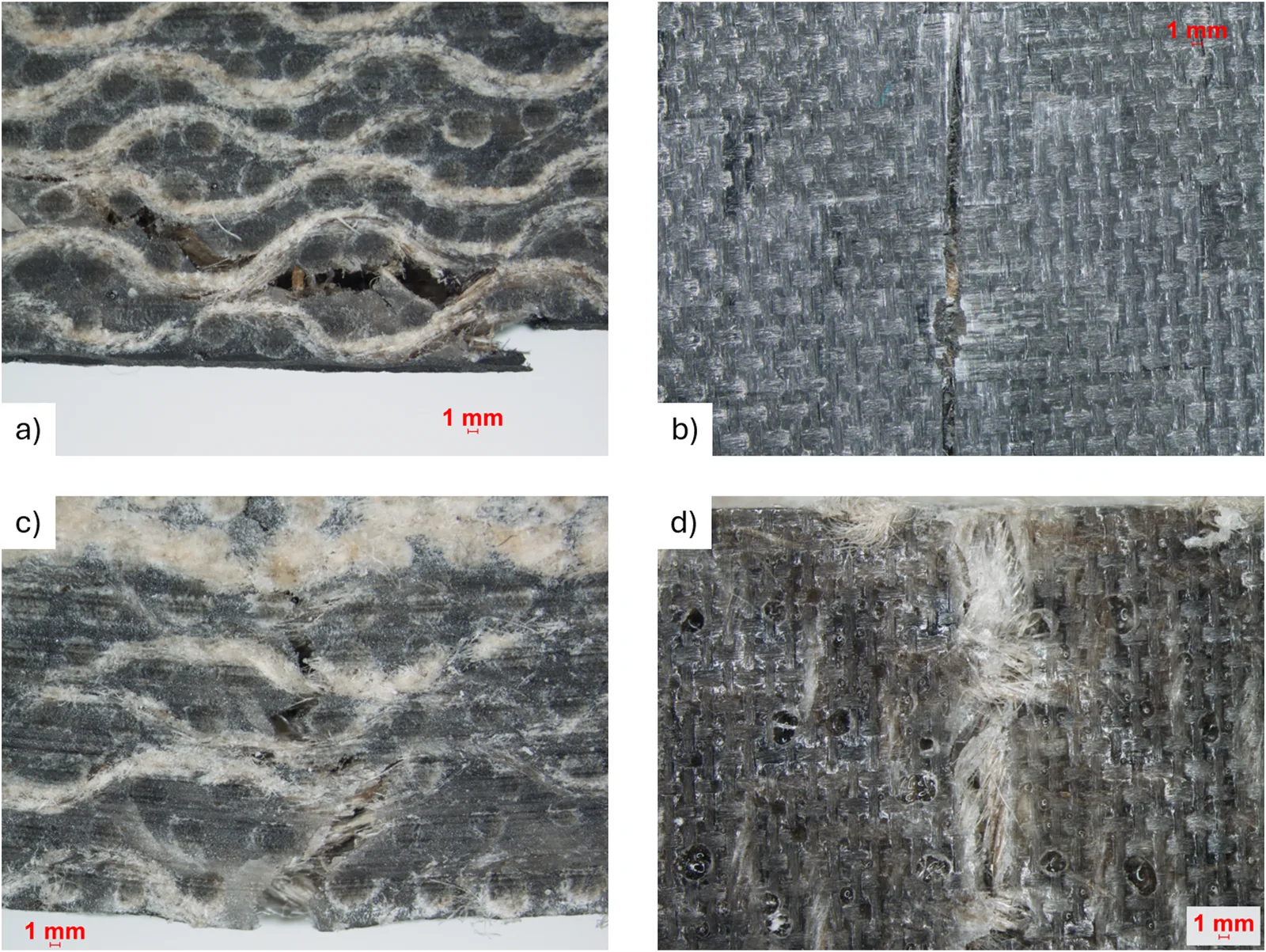
Failure images for flax and carbon-flax specimens showing a variety of views following short beam tests including a) Carbon-Flax Side View, b) Carbon-Flax Bottom View, c) Flax Side View and d) Flax Bottom View.

### Tensile testing results

The average OHT strength for the flax specimens was 54.1 (± 1.3) MPa while the addition of carbon facing plies increased this by only 0.5% to 54.4 (± 1.6) MPa. The difference in unnotched tensile strength was similarly small (0.1%) with an average strength of 70.4 (± 1.36) MPa for the flax specimens and 70.5 (± 2.5) MPa for the carbon-flax specimens. Full stress–strain plots for flax and carbon-flax specimens obtained from OHT testing are provided in Figure 8. All OHT and unnotched tensile specimens failed in the Lateral Gage Middle (LGM) failure mode except for unnotched flax specimen five which failed in the Angled Gage Middle (AGM) mode. Exemplar images of failure modes across specimens are shown in Figure 9.

**Figure 8.**
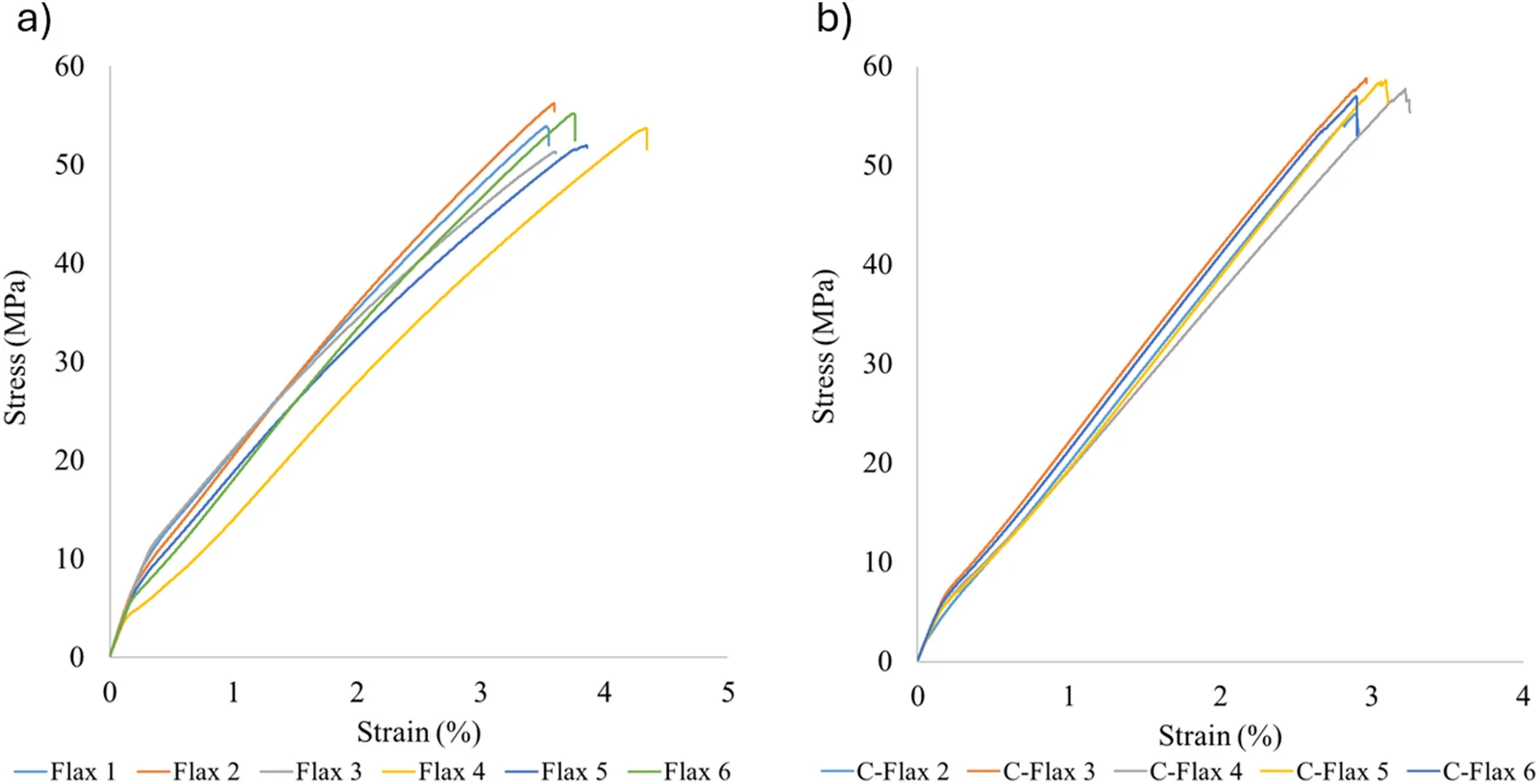
Stress–strain plots from OHT testing for (a) flax and (b) carbon-flax specimens.

**Figure 9.**
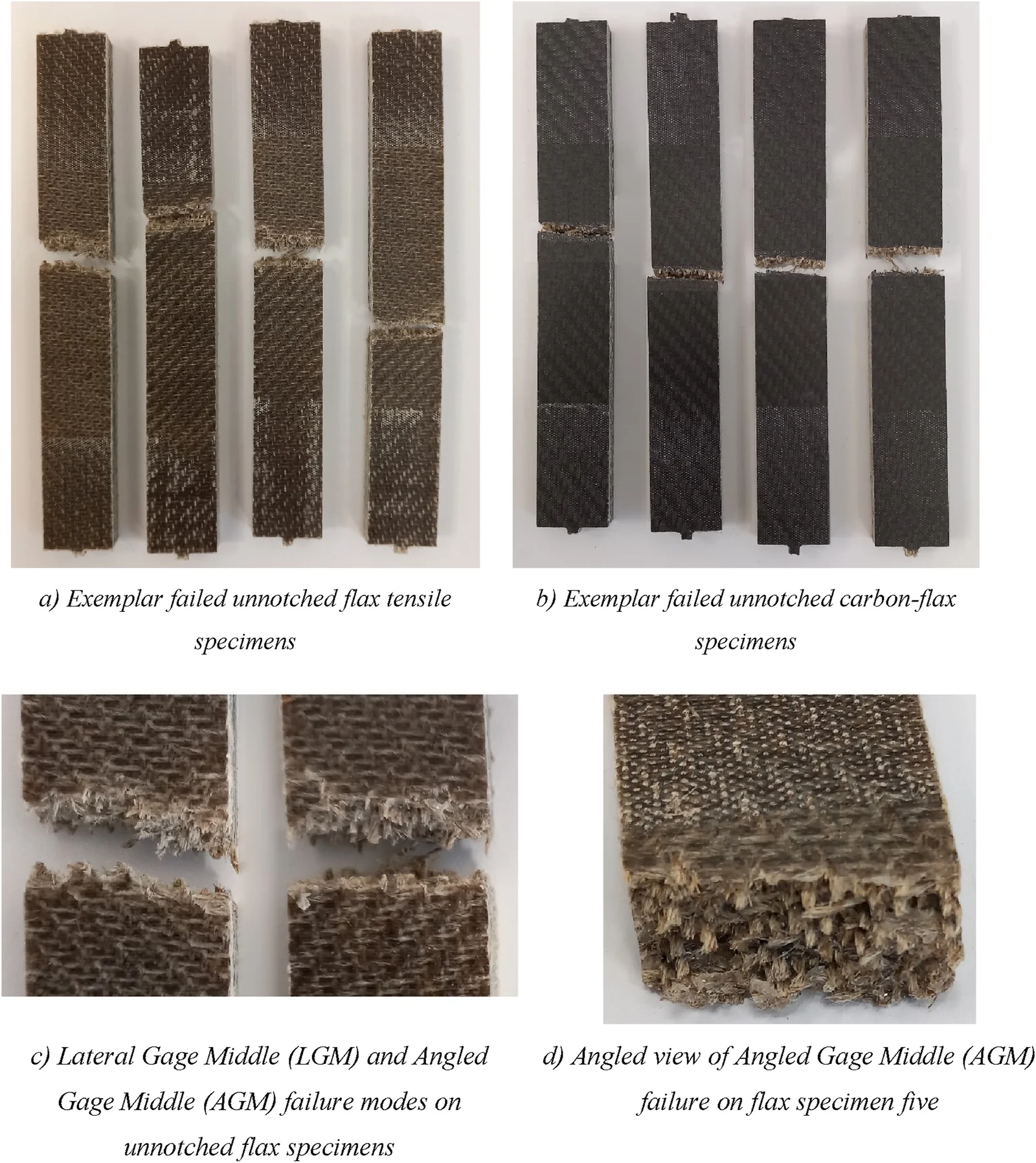
Images of failed unnotched tensile specimens.

Although the unnotched tensile strengths of flax and carbon-flax hybrid laminates were nearly identical, this behaviour is consistent with the mechanics of thick hybrid laminates where the majority of the axial load is carried by the interior plies. In the present 1-8-1 lay-up, the two carbon face plies represent only a small fraction of the total cross-section (∼5%). Since the tensile test produces a nearly uniform axial stress through the thickness ( $\sigma_{i}=E_{i}\varepsilon$ ), the stiffer carbon layers do not dominate the net-section strength; instead, failure initiates within the thick flax core, which governs the load-bearing capacity of the laminate. Using the approximate elastic moduli of the two constituent fibres $E_{flax}=8\;GPa$ and $E_{carbon}=230\;GPa$ , the carbon face plies experience approximately twenty-nine times higher axial stress than the flax plies at the same applied strain. However, carbon fibres typically fail at lower strains than woven flax fibres. As such, the carbon plies reach their failure strain much earlier during loading. Although $E_{carbon}$ is substantially larger than $E_{flax}$ , the small area fraction $A_{carbon}\ll A_{flax}$ limits the contribution of the carbon plies to the total tensile force. Consequently, failure is governed by the thick flax core and explains why the experimentally measured unnotched tensile strengths of the flax and carbon-flax laminates are nearly identical, despite the large stiffness contrast between the constituent materials. The OHT strength Knock-Down Factor (KDF) ( $\sigma_{open-hole}/\sigma_{unnotched}$ ) was 0.77 for both laminate types, reinforcing the influence of the central flax core.

Hybridisation therefore increases stiffness – reflected in the 66% increase in flexural modulus and improved ILSS – but has limited influence on ultimate tensile strength, which is controlled by the larger proportion of flax material. Specifically, for OHT, strength remains similar between the two systems, as the presence of a hole further shifts the load distribution toward the inner flax plies. Surface carbon plies exhibit reduced influence on failure initiation around the hole. Hybridisation therefore affects stiffness more strongly than strength in this configuration, explaining the flexural and ILSS improvements without corresponding increases in tensile or OHT strength.

Figure 10 shows SEM images of two points on the tensile failure surface where there are many broken flax fibres. These images suggest that the mode varies across the failure surface. For example, the left image presents like a disordered fracture that may improve toughness, whereas the failure on the right appears more brittle in nature.

**Figure 10.**
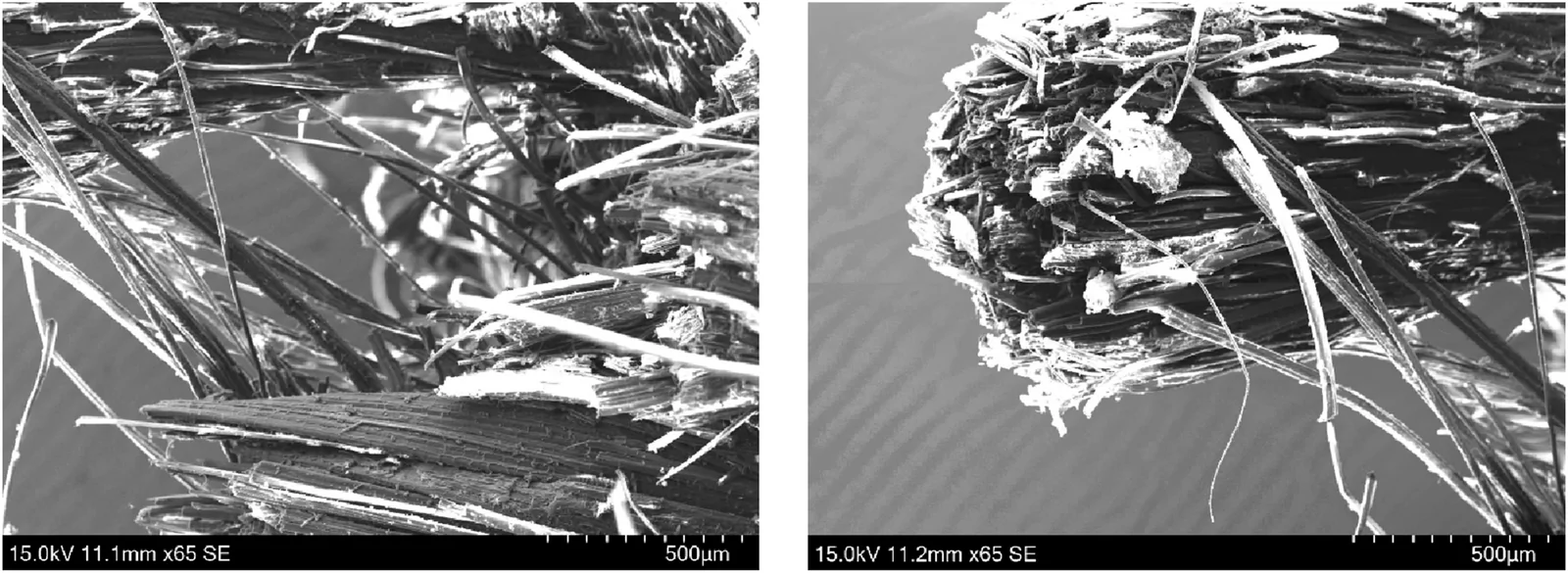
Failure surface SEM images after unnotched tensile testing.

DIC in-plane strain fields were captured during loading to understand the damage progression in the different specimens. Figure 11 shows the in-plane engineering strains along the loading direction (ε_yy_) measured at four stress levels of 0.25 σ_UTS_, 0.5 σ_UTS_, 0.75 σ_UTS_ and at the point of failure, in a localised region around the hole. Higher strains were observed at increasing stress levels and the expected stress concentration effect occurred around the hole. Strain localisation was observed around the hole in all samples; however, this was higher and more pronounced for the flax only specimens Figure 11(a). It can be seen that the stress concentration around the hole for the flax specimen has reached 0.0225 microstrain at 0.50 σ_UTS_ but this only occurs at 0.80 σ_UTS_ for the carbon-flax specimen. As such, the flax laminates exhibit earlier and more severe strain localisation, with pronounced gradients developing at lower fractions of σ_UTS_. Results show that the external carbon plies have an effect on the regularisation of the strain fields and structural performance, that is, the stiffer outer plies reduce surface strain amplification, delaying localisation near the hole, and a more spatially uniform strain field. Higher strain gradients near the hole and different strain concentrations are observed for the flax only specimens. For example, in the flax specimens, at 0.75 σ_UTS_ and 0.99 σ_UTS_ a pattern of ε_yy_ (loading direction) strain peaks are observed on the specimen surface. This illustrates the load carrying role of the carbon outer plies - also seen in both the ILSS samples – where the failure is transverse (potentially due to resin cracks) for the flax laminate but interlaminar (fibre/matrix interface failure) for the hybrid lay-up. While the presence of outer carbon plies doesn’t significantly change the peak strength, it delays the strain-to-failure.

**Figure 11.**
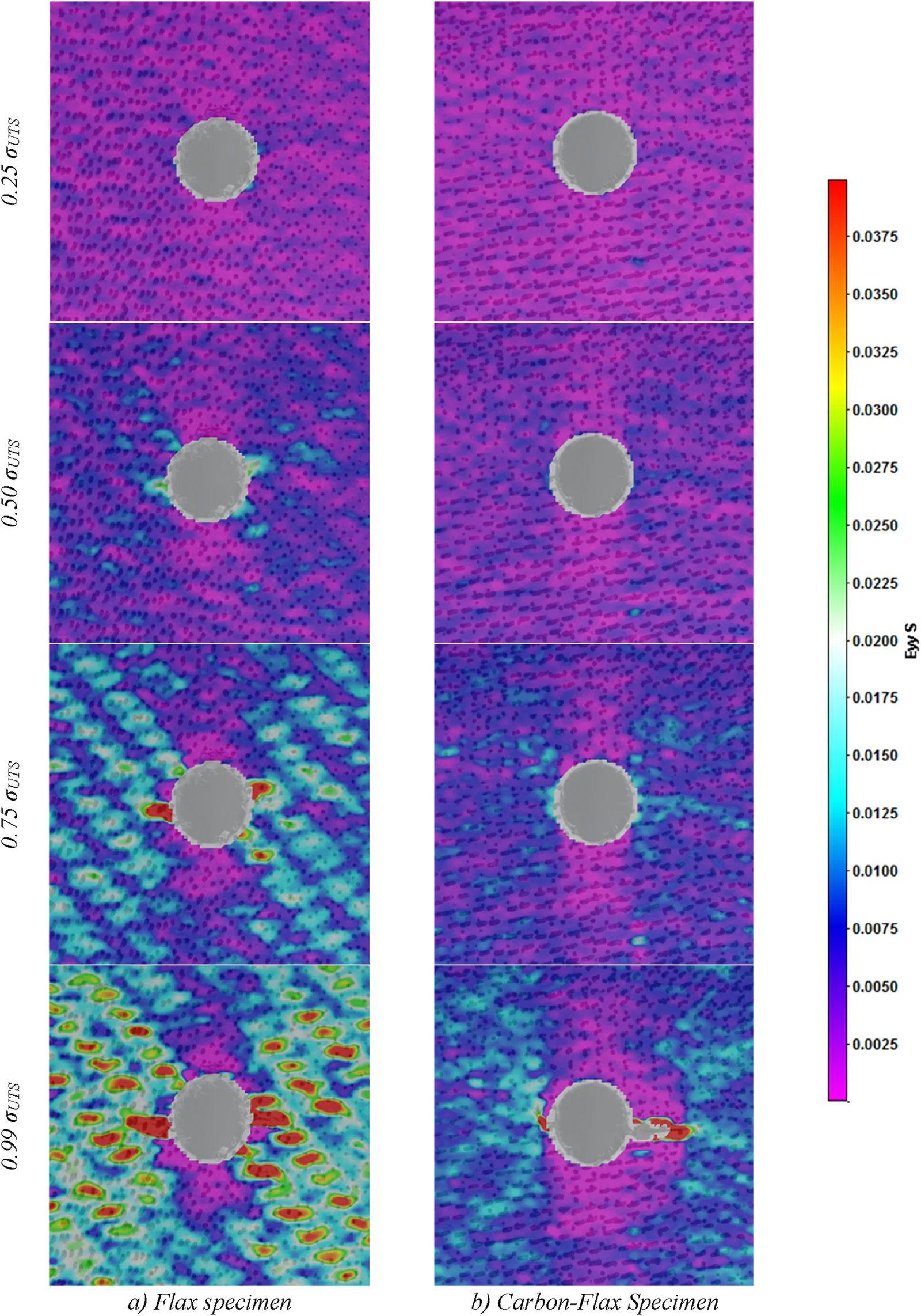
Comparison of εyy (microstrains) strain distribution showing localisation near the hole at increasing nominal stress levels for (a) flax and (b) carbon-flax specimens. The colour map corresponds to engineering strain εyy obtained via DIC at a subset size of 29 pixels.

Full field axial and shear strain maps and plots of strain versus distance from hole for flax specimens are shown in Figure 12. All strain maps were extracted at 50 MPa load to allow comparison before failure. Like the observations of Hawkins et al.,^34^ the median strain plots do not capture the peak strain concentrations that would typically be expected at the smallest radial distance from the hole. At a global stress of 50 MPa, the full-field strain maps show distinct localisation behaviour that is strongly dependent on the laminate configuration. For the flax-only specimens, the axial strain field develops higher gradients and multiple localisation bands extending from the hole, indicating earlier strain redistribution and local damage initiation within the surface plies. In comparison, the hybrid laminate exhibits a more regularised strain field, demonstrating that the carbon facesheets suppress out-of-plane deformation and delay strain accumulation. The peak shear strains extend from the hole in an approximately diagonal direction, reflecting the orthotropic stiffness of the woven architecture. Plots of median strain, Figure 12(c) and (d), indicate that the stress concentration around the hole is significantly larger than at the free edges of the specimen, which is consistent with the small variance in OHT strength across flax specimens.

**Figure 12.**
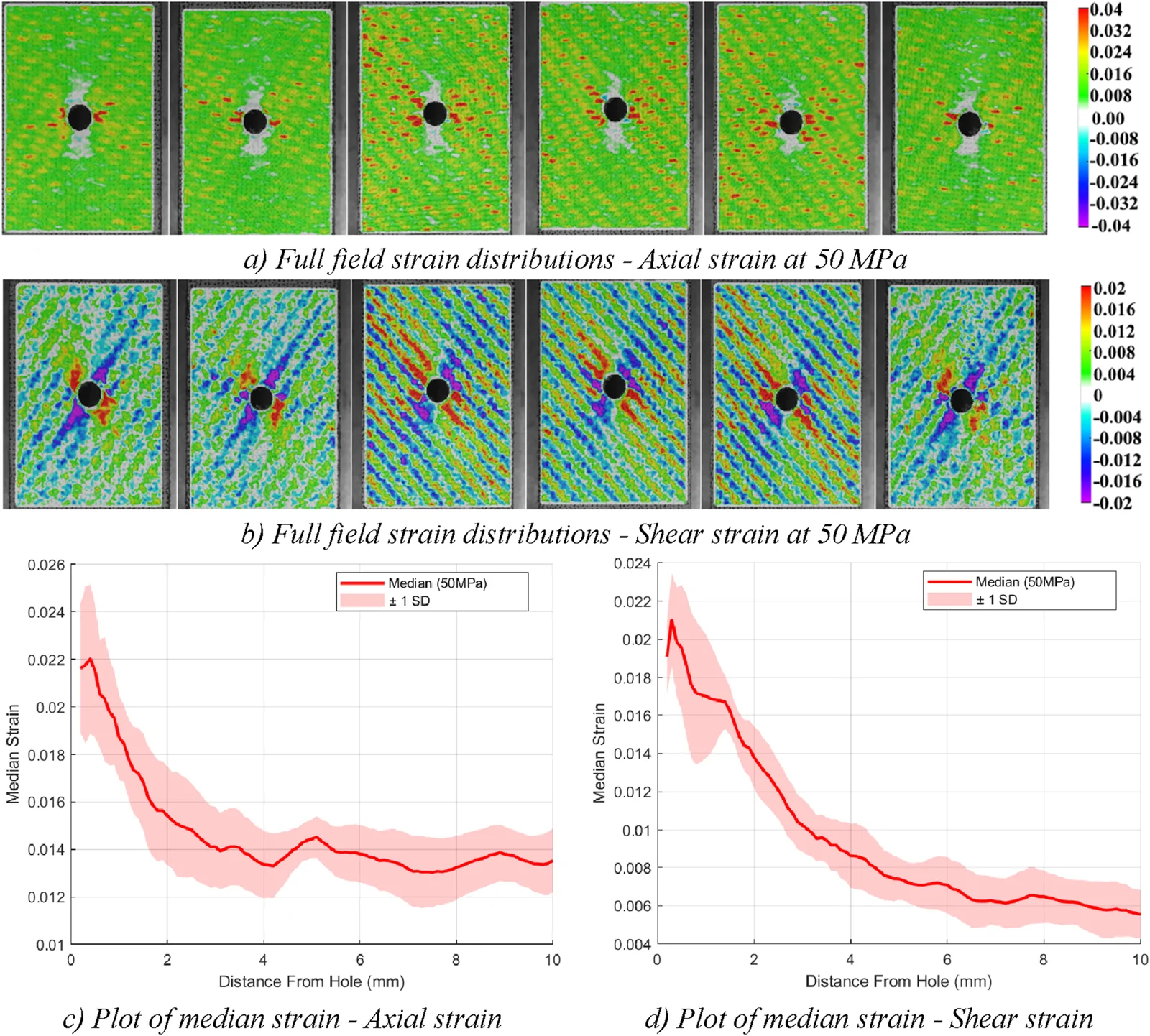
OHT full field strain distributions and corresponding plots of median strain at radial distance from the hole for flax only specimens.

Although both material systems displayed the expected stress-concentration effect around the hole, the hybrid laminate maintained a more distributed strain pattern until higher loading. This demonstrates a difference in load sharing and damage initiation mechanisms: the hybrid architecture suppresses early localisation, whereas the flax-only laminate accumulates strain more rapidly near the notch, which is consistent with the more rapid emergence of local damage.

It should be noted that since DIC measures strain only on the outer laminate surface, the observed regularisation of strain gradients in the hybrid specimens predominantly reflects the behaviour of the carbon face plies and outer-ply stiffness effects that do not necessarily fully penetrate the 10 mm laminate thickness. As such, these 2D DIC results are not likely to reflect the complex the strain state the interior flax plies experience (∼80% of the laminate thickness). As such, hybridisation influences surface strain evolution and since failure initiates within the flax core, improved surface strain regularisation does not directly translate into increased open-hole tensile strength and does not substantially alter through-thickness stress redistribution. This also helps to explain why DIC maps show delayed localisation in the hybrid specimens, while the OHT strength remains nearly unchanged. In thick laminates, improvements in surface strain distribution may delay surface damage but are insufficient to suppress failure mechanisms originating in the laminate core. As such, strength remains governed by internal damage evolution rather than surface strain localisation alone.

### Combined loading compression testing results

The average CLC strength for the flax specimens was 72.9 (±0.84) MPa while the addition of carbon facing plies increased this by 5.8% to 77.1 (±0.77) MPa. The failure mode of both specimen types was Brooming Gage Middle (BGM) as shown in Figure 13, and all specimens failed in the guage section. This failure mode arises from progressive kink-band formation and matrix microcracking and fibre splitting, rather than brittle instability, under combined end loading and shear. This is a physically meaningful failure mode in thick laminates under CLC as the brooming-type failures occurred within the gauge section and were not triggered by fixture contact or end crushing. Importantly, the repeatability of the measured compressive strengths, low standard deviation, and the consistency of failure morphology across flax and carbon-flax specimens indicate that brooming did not introduce uncontrolled scatter or invalidate the results. Instead, it represents the dominant physical failure mechanism governing compressive performance in these materials. The slight improvement in CLC strength in the hybrid laminate is attributed to increased bending stiffness from carbon faces, which delays surface microbuckling but does not prevent core-driven brooming failure.

**Figure 13.**
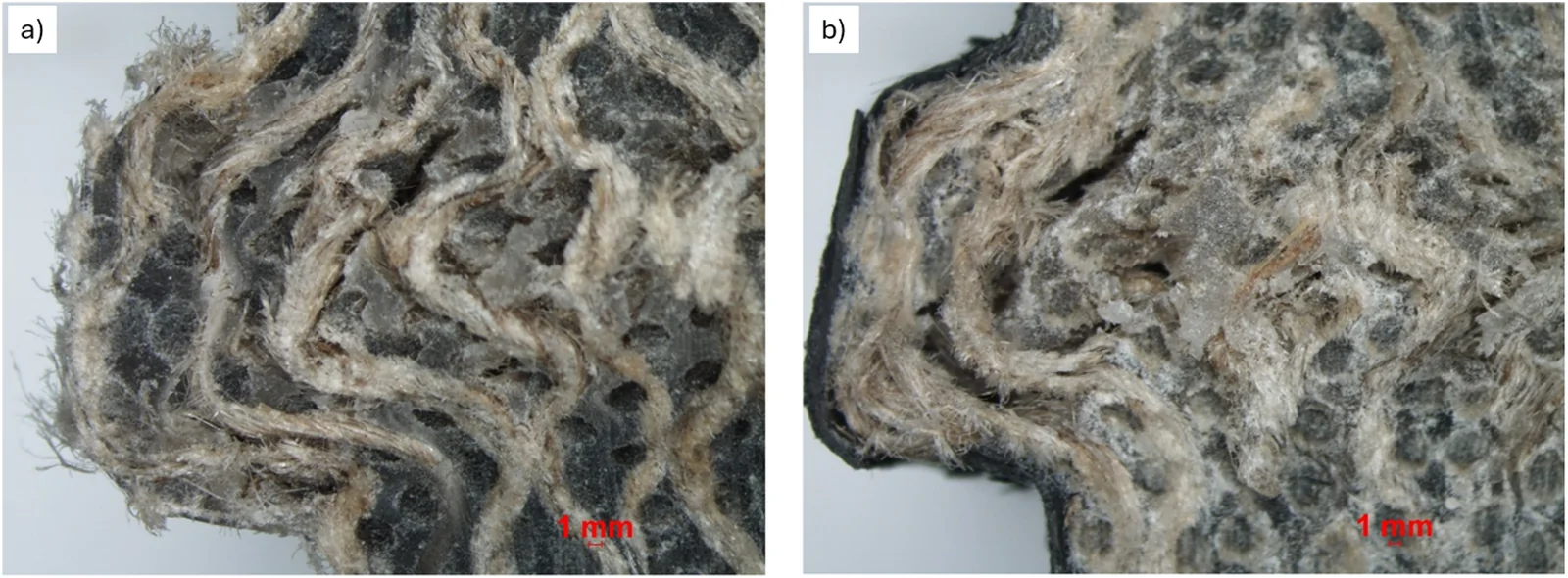
Microscope images of CLC failure modes in (a) flax and (b) carbon-flax specimens showing Brooming Gage Middle (BGM) failure mode.

### Flexural testing results

The flexural strength for each specimen was calculated using equation (2):

(2) $$\sigma_{f}=3PL/2bd^{2}$$

where $\sigma_{f}$ is the stress at the midpoint (MPa), P is the load at a given point on the load-deflection curve (N), L is the support span, and b and d are the specimen width and depth, respectively, all in millimetres. Figure 14 compares the force-displacement plots for flexural testing on both flax and carbon-flax laminates. The average flexural strength for the flax specimens was 99.6 (±4.9) MPa while the addition of carbon facing plies increased this by a similar amount to ILSS (25%) to 124.2 (±5.35) MPa. The slope of the carbon-flax specimens was significantly steeper than the flax specimens. As such, the average flexural modulus ( $E_{B}=L^{3}m/4bd^{3}$ ) for the flax specimens was 7.65 (±0.31) GPa but increased by 66% to 12.7 (±0.55) GPa with the addition of carbon facing plies. This further supports the observation from tensile/OHT testing that hybridisation enhances stiffness, not strength. Flexural stiffness was dominated by the outer plies and can be quantitatively explained using sandwich beam theory. In the present 1-8-1 laminate (total thickness ∼10.36 mm), the two carbon face plies together represent less than 5% of the cross-section but are located at the maximum distance from the neutral axis (∼4.9 mm). The cores contribution to bending stiffness can be estimated using $D_{c}=\frac{E_{c}bc^{3}}{12}$ , where $E_{c}$ is the modulus of the core (Pa), b is the beam width (mm), and c is the core thickness (mm). Since these hybrid laminates have stiff faces ( $E_{f}$ ) and a comparatively weak core, the effective bending stiffness of the sandwich can be determined using $D_{eff}=\frac{E_{f}btc^{2}}{2}$ where t is the face thickness (mm) and $E_{f}$ is the modulus of the facesheet (Pa).^35^ Noting the approx. elastic moduli of the two constituents, despite accounting for less than 5% of the total thickness, the carbon face plies contribute over 80% of the total flexural rigidity. Although the facesheets are thin, their higher modulus and placement at a large distance from the neutral axis dominate the bending response. This explains why only two outer carbon plies results in a 66% increase in measured flexural modulus. The average displacement at break for the carbon-flax specimens was 7.8 (±0.6) mm which was 40% smaller than the flax specimens, 12.90 (±0.99) mm. Carbon face plies carry most of the bending load, dramatically increasing modulus and reducing displacement at break. This behaviour contrasts with tensile behaviour, where the flax core governs performance.

**Figure 14.**
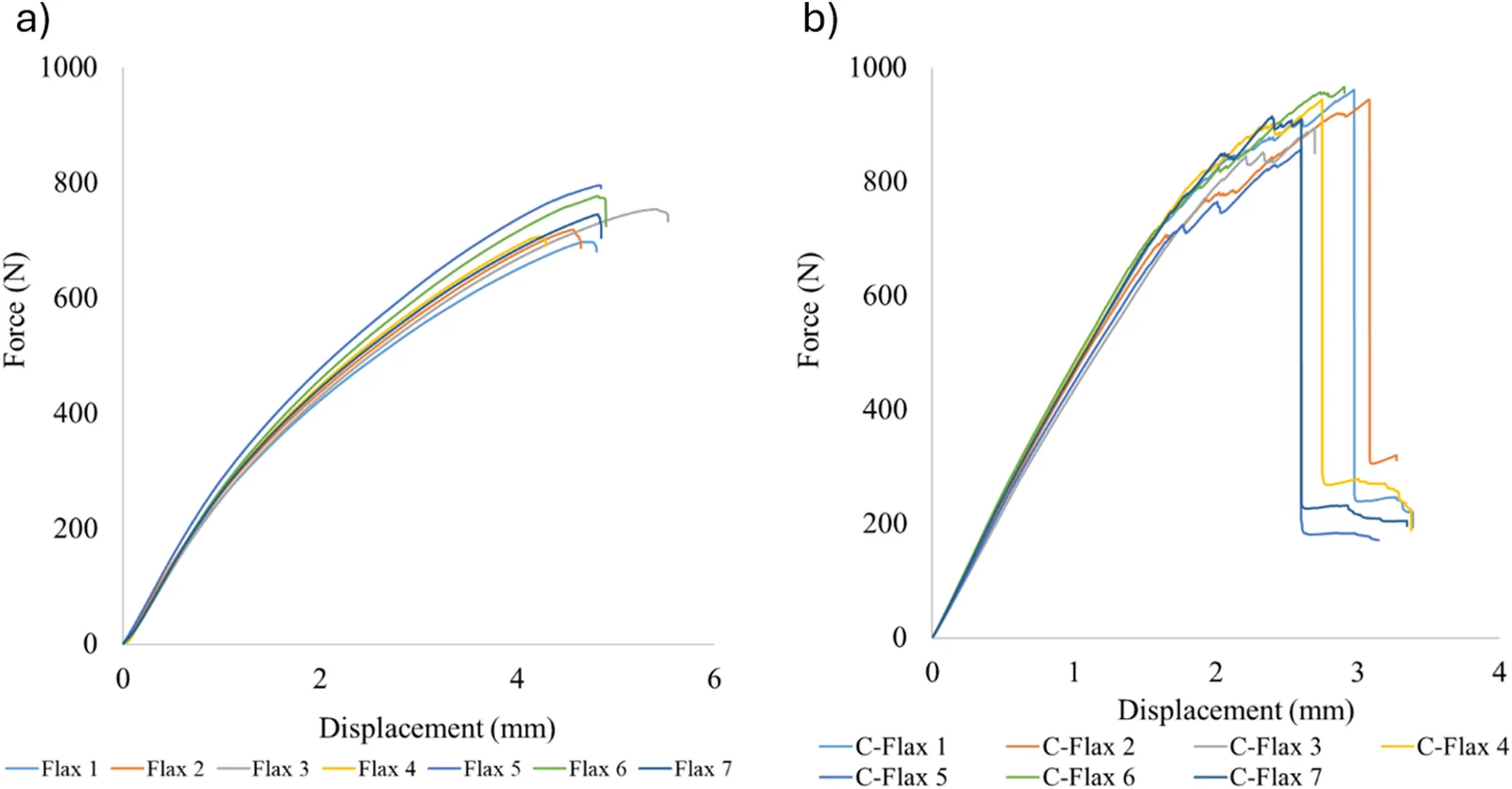
Force–displacement plots for flexural testing on (a) flax and (b) carbon-flax laminates.

The above interpretation of flexural stiffness is further supported by the measured increase in interlaminar shear strength. The improved ILSS demonstrates enhanced resistance to interfacial shear failure, which is essential for effective stress transfer in bending-dominated loading. If interlaminar shear transfer were inadequate, one would expect a smaller-than-predicted increase in flexural modulus, premature shear-driven delamination and significant non-linearity in the force-displacement response. None of these behaviours were observed experimentally. Instead, the agreement between flexural stiffness enhancement and ILSS improvement confirms that the carbon face plies are well-coupled to the flax core and actively contribute to the laminate’s bending response.

### Summary of results and implications

This work attempted to better understand the structure-property relationships governing failure in thick flax laminates across different loading modes. The key contribution of this work is a comparative assessment of flax and carbon-flax hybrid laminates across a range of tensile, open-hole tensile (with DIC), flexural, interlaminar shear (ILSS) and combined loading compression tests on thick (∼10 mm) laminates. The results provide new insight into the mechanical behaviour of these laminates and the influence of carbon face-ply hybridisation. Most significantly, hybridisation selectively enhances stiffness- and interface-dominated properties (flexure, ILSS), while exerting minimal influence on net-section tensile and OHT strength. The hybrid laminate’s unnotched tensile and OHT strengths differ from pure flax by less than 1%. In contrast, under flexural loading, outer plies dominate stiffness due to their distance from the neutral axis; thus, the hybrid shows a 66% increase in flexural modulus despite minimal changes in tensile strength, while ILSS increased by 29% with hybridisation.

Microscopy showed that flax laminates failed largely through vertical tow splitting, whereas hybrid laminates exhibited interlaminar shear cracking along diagonally oriented planes near the carbon/flax interface. This reflects the mechanical interplay between stiff carbon plies restraining deformation and redistributing interlaminar stresses. These findings align with prior observations of enhanced interlaminar behaviour in flax-carbon hybrid systems, but this study demonstrates the effect in thick laminates.

DIC revealed that hybrid laminates exhibit delayed strain localisation around open holes, producing smoother gradients on the outer surface. The hybrid’s improved surface strain distribution does not significantly influence OHT strength because failure initiates within the flax core. This helps reconcile the counterintuitive observation that improved near-hole strain fields do not translate to strength increases. In CLC testing, both laminate types exhibited brooming-type failure. The modest 5.8% increase in compression strength for the hybrid laminate indicates that carbon face plies delay surface buckling but do not prevent the formation of kink bands deep within the flax core. This reinforces the conclusion that hybridisation’s benefits are strongest where face plies dominate the mechanical response.

The findings of this work can have implications for the design of natural-fibre hybrid laminates, highlighting key considerations for the design of thick natural-fibre laminates:

• Hybridisation is effective when outer plies dominate the mechanical response (e.g. flexure and interlaminar shear),
• Strength-driven applications may require higher carbon fractions or repositioning of carbon layers deeper into the laminate,
• Thick natural-fibre laminates exhibit failure mechanisms that cannot be extrapolated from thin-laminate data.

Table 1 provides a summary of the mechanical properties across samples and tests.

**Table 1.** Summary of mechanical properties across samples.

		Flax	Carbon-flax
Laminate tensile modulus	Unnotched (MPa)	4.5 ± 2.8	4.0 ± 1.5
Laminate tensile strength	Unnotched (MPa)	70.4 ± 1.4	70.5 ± 2.5
	OHT (MPa)	54.1 ± 1.3	54.4 ± 1.6
Laminate compressive modulus	$E^{C}$ (MPa)	103.7 ± 11.5	118.2 ± 20.2
Laminate compressive strength	$F^{cu}$ (MPa)	72.9 ± 0.8	77.1 ± 0.8
Interlaminar shear strength	$\tau_{12}$ (MPa)	13.1 ± 0.5	16.8 ± 0.3
Modulus of elasticity in bending	$E_{B}$ (MPa)	7.7 ± 0.3	12.7 ± 0.6
Flexural strength	$\sigma_{f}$ (MPa)	99.6 ± 4.9	124.19 ± 5.4

## Conclusions

This work assessed the structure-property relationships governing failure in thick (∼10 mm) flax fibre reinforced polymer (FFRP) laminates across different loading modes, using unnotched tensile, combined loading compression (CLC), open-hole tensile, flexural and interlaminar shear strength (ILSS) tests. Additional laminates, with Carbon Fiber Reinforced Polymer (CFRP) facesheets in a 1-8-1 arrangement, were also tested to examine the influence of face-ply hybridisation on mechanical behaviour. The results provide new insight into the mechanical behaviour of these laminates and the influence of carbon face-ply hybridisation. The most significant finding is that hybridisation selectively enhances stiffness- and interface-dominated properties (flexure, ILSS), while exerting minimal influence on net-section tensile and OHT strength. Based on the results of this investigation, the following conclusions can be drawn:

• Across all specimens, results were consistent among tests, that is, carbon-flax hybrid laminates generally exhibited higher stiffness and interlaminar performance than flax-only laminates,
• Unnotched carbon-flax specimens exhibited similar tensile strength to flax-only laminates, indicating that tensile failure remains governed by the thick flax core,
• For flax specimens, the average ILSS was 13 MPa, average OHT strength was 54.1 MPa, average unnotched tensile strength was 70.4 MPa, average CLC strength was 72.9 MPa and the average flexural strength was 99.6 MPa,
• The addition of carbon-fibre facing plies had a modest effect on strength properties except for ILSS and Flexural strength, which increased by 29% and 66%, respectively, due to the dominant role of outer plies in these loading modes,
• Digital Image Correlation (DIC) showed earlier and more pronounced strain localisation in flax-only specimens, while hybrid laminates exhibited delayed surface strain localisation,
• Despite improved surface strain distribution, hybridisation did not increase OHT strength, suggesting that failure initiation remains governed by interior plies,
• Compression behaviour in both laminate systems was controlled by internal kink-band formation – both laminates failed via brooming-type modes; hybridisation provided only modest increase in CLC strength,

These findings provide further details and understanding on the mechanical and structural behaviour of thick FFRP laminates and highlight the importance of laminate architecture when applying hybridisation strategies. Future studies could focus further on assessing the manufacturing of these types of laminates, paying close attention to infusion quality and the presence or absence of microstructural defects such as porosity. Additionally, examining the thermal behaviour (thermal-expansion) of natural-carbon fibre hybrid materials and identifying other materials to partner with flax in a hybrid could make FFRP laminates an attractive option for future composite applications.

## Data Availability

Experimental details are discussed in detail in the manuscript. The data supporting the findings of this study are available from the corresponding author upon reasonable request.*
